# Multiscale network modeling reveals the gene regulatory landscape driving cancer prognosis in 32 cancer types

**DOI:** 10.1101/gr.278063.123

**Published:** 2023-10

**Authors:** Peng Xu, Bin Zhang

**Affiliations:** 1 Department of Genetics and Genomic Sciences, Icahn School of Medicine at Mount Sinai, New York, New York 10029, USA;; 2 Mount Sinai Center for Transformative Disease Modeling, Icahn School of Medicine at Mount Sinai, New York, New York 10029, USA;; 3 Icahn Institute for Data Science and Genomic Technology, Icahn School of Medicine at Mount Sinai, New York, New York 10029, USA;; 4 Department of Pharmacological Sciences, Icahn School of Medicine at Mount Sinai, New York, New York 10029, USA

## Abstract

Cancer is a complex disease with diverse molecular mechanisms that affect patient prognosis. Network-based approaches are effective in revealing a holistic picture of cancer prognosis and gene interactions. However, a comprehensive landscape of coexpression networks and prognostic gene modules across multiple cancer types remains elusive. In this study, we performed a systematic analysis of coexpression networks in 32 cancer types. Our analysis identified 4749 prognostic modules that play a vital role in regulating cancer progression. Integrative epigenomic analyses revealed that these modules were regulated by interactions between gene expression and methylation. Coregulated genes of network modules were enriched in chromosome cytobands and preferentially localized in open chromatin regions. The preserved network modules formed 330 module clusters that resided in chromosome hot spots. The cancer-type-specific prognostic modules participated in unique essential biological processes in different cancer types. Overall, our study provides rich resources of prevalent gene networks and underlying multiscale regulatory mechanisms driving cancer prognosis, which lay a foundation for biomarker discovery and therapeutic target development.

Cancer is a multifaceted and intricately regulated process that involves the coordinated activity of genes from various pathways. Despite the diversity of cancer types, there exists a common framework that governs their development and progression (Hanahan and Weinberg 2011). Various network approaches have been proposed to characterize gene interactions and dissect the regulatory relationships underlying tumorigenic pathways (Creixell et al. 2015). Gene coexpression networks are a mathematical model that assumes functionally related genes are coregulated as a cellular system and can be modeled as modules embedded in a coexpression network (Zhang and Horvath 2005; Langfelder and Horvath 2008). Characterizing gene coexpression networks is vital for cancer research, as they provide a holistic view of how genes function together in biological systems, allowing identification of novel gene interactions and pathways.

Discovering genes that predict patient survival is crucial for cancer biomarker development, risk assessment, and clinical decision-making. Previous studies showed that cancer prognostic genes tend to be coregulated at the transcriptome level and enriched in the modules of coexpression networks (Yang et al. 2014). As coexpression modules reflect holistic features of pathway-related genes, they are robust and meaningful for cancer prognosis and functional interpretations. Multiple computational methods have been applied to identify cancer networks and prognostic modules. For example, Yang et al. (2014) detected network modules enriched for prognostic genes in four cancers using our previously developed tool WGCNA (Zhang and Horvath 2005). Yu et al. (2019) focused on seven cancer types and constructed a network using the top correlated gene pairs, followed by community detection via MCODE. Raina et al. (2023) established a database of coexpression networks from TCGA data sets using the weighted Pearson correlation method, without subsequent modularity and prognostic marker identification. Although prior research has identified coexpression network modules in a few cancers, a standard procedure for defining such networks across a broad range of cancer types remains absent.

In this study, we used the advanced, uniform, and well-regarded network tool multiscale embedded gene coexpression network analysis (MEGENA) to systematically identify prognostic modules. In contrast to the large, sparse modules created by WGCNA (Zhang and Horvath 2005), MEGENA has been empirically verified using simulated and real-world large-scale gene expression data to resolve intricate hierarchical network structures and generate more concise and coherent modules (Song and Zhang 2015; Chella Krishnan et al. 2018). The high resolution of hierarchical network structures at varying levels of compactness allows MEGENA to efficiently infer coexpression relationships and further pinpoint network drivers in large-scale bulk tissue transcriptomes of complex human diseases (Wang et al. 2019, 2021; Song et al. 2021; Xu et al. 2022). By applying MEGENA to all major cancer types within the TCGA cohort, we comprehensively characterized coexpression networks and prognostic modules in cancers and revealed the multiomic regulatory mechanisms related to cancer prognosis. We also shared all the networks, modules, prognostic features, and functional annotations on open-source websites, which offers a unique opportunity for comparative network analysis in this field.

## Results

### Pan-cancer coexpression networks and prognostic modules

**Figure 1.**
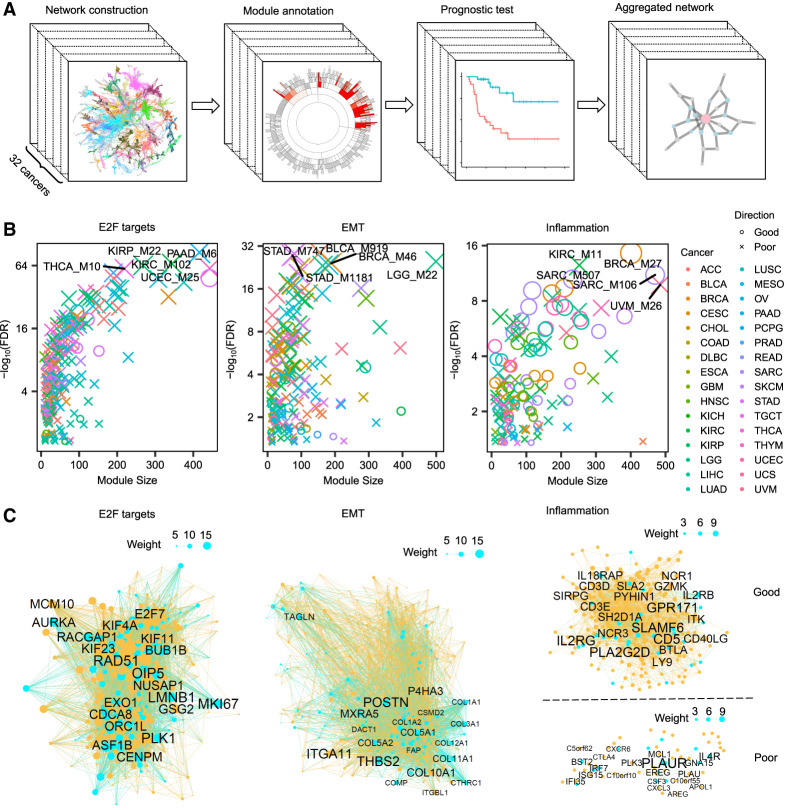
Pan-cancer coexpression networks and prognostic modules. (*A*) The pipeline for pan-cancer coexpression network analysis, showing the steps involved in constructing coexpression networks and identifying prognostic modules. (*B*) Dot plots of three representative hallmark pathways of prognostic modules. For each pathway, the dots indicate the prognostic modules from 32 cancers, and the colors show the outcomes of patient survival in each cancer. The dot size is proportional to the pathway enrichment significance, with larger dots indicating stronger enrichment. The false-discovery rate (FDR) was calculated as the adjusted Fisher's exact test (FET) *P*-values. The top five modules with the strongest enrichment are labeled. (*C*) Aggregated networks of the prognostic modules in the three hallmark pathways, with blue dots indicating the pathway genes and yellow dots indicating other coexpressed genes. The node size in each network is proportional to the conservation weight across different cancers. The top 20 nodes with the strongest conservation are labeled.

We constructed gene coexpression networks for 32 solid tumors using the MEGENA pipeline, which includes 9546 individuals from the TCGA database. We performed four major analyses, including gene network construction, gene module annotation, module prognostic testing, and module preservation analysis (Fig. 1A). MEGENA ranks significant correlation coefficients and iteratively tests them for planarity to grow a planar filtered network using the PMFG algorithm (Tumminello et al. 2005). Then it performs multiscale clustering analysis to identify coexpression modules at different network-scale topologies. The modules generated by MEGENA coexpression networks are hierarchical, ranging from the smallest modules with 10 genes to large ones with thousands of genes. In total, MEGENA identified 27,448 hierarchical modules from 32 coexpression networks corresponding to 32 different cancer types (Supplemental Table S1). On average, each network contains 17,831 genes (SD = 1568), 51,677 gene–gene correlations (SD = 1487), and 858 hierarchical network modules (SD = 139).

The pan-cancer network analysis identified 4749 prognostic modules, whose module eigengenes (PC1 from principal component [PC] analysis [PCA]) were correlated with survival outcomes of cancer patients from the Cox proportional-hazard model (*P* < 0.05) (Supplemental Table S2). Based on Molecular Signatures Database (MSigDB) hallmark pathway annotation, the prognostic modules were most enriched for pathways essential for tumor development, including E2F targets, G2M checkpoints, epithelial–mesenchymal transitions (EMTs), MYC targets, and inflammatory responses (Supplemental Fig. S1). Of the 239 prognostic modules enriched for the E2F target pathway, 205 (86%) modules were associated with poor survival or a higher hazard ratio (Fig. 1B,C). Similarly, 184 of 209 (88%) prognostic modules of the EMT pathway showed poor survival. Meanwhile, about half of (50%, n = 77) the prognostic modules of the inflammatory pathway showed good survival, and another half of the inflammation-related modules corresponded to poor survival. We also observed that there were more modules associated with lower survival than with increased survival (Supplemental Fig. S1). Given that the pathways of E2F targets and EMT were conserved across multiple cancer types, there were more prognostic modules enriched for these two pathways linked with poor survival. Other pathways, such as fatty acid metabolism, adipogenesis, and xenobiotic metabolism, were associated with a good survival rate and were more specific to certain cancer types.

We evaluated the predicting efficacy of prognostic modules in comparison to prognostic genes. Given that the eigengene of prognostic modules was calculated from PC analysis, we extracted all the PCs of prognostic genes for comparison with the prognostic modules. In breast cancer (BRCA), there were 1670 prognostic genes, and only 46 PCs of the prognostic genes were associated with survival (*P* < 0.05), which is fewer than the number of prognostic modules (n = 103). Among the top 50 ranked PCs, prognostic modules also show a higher significance than prognostic genes in predicting patient survival (Wilcoxon test *P* = 1.9 × 10^−6^) (Supplemental Fig. S2). These findings indicate that the module-based approach is more effective at identifying survival-associated features than is individual gene analysis. Because the network modules represent all individual genes in a module, the network approach is able to unveil interactions of tumor regulators. For example, two key cytolytic effectors, granzyme A (*GZMA*) and perforin 1 (*PRF1*), were coexpressed in the aggregated inflammation network associated with good patient survival (Supplemental Table S3). Consistently, the combination of these two genes was effective to quantify immune cytolytic activity in several cancers (Rooney et al. 2015).

To investigate whether network modules could be reproduced in non-TCGA cohorts, we analyzed RNA sequencing data from tumor tissues of 65 liver cancer patients (LIHC) in an independent study (Long et al. 2022). We used the same MEGENA pipeline to construct a coexpression network and evaluated the conservation of TCGA network modules in this cohort. Of the 796 network modules identified in the LIHC from the TCGA data set, 661 (83%) were recovered in the non-TCGA cohort (adjusted Fisher's exact test *P* [aFET*P*] < 0.05) (Supplemental Table S4). Similarly, 199 out of 237 (84%) prognostic modules from TCGA were preserved in the independent cohort. We also explored whether crucial functions of pan-cancer prognostic modules could be reflected in cell lines. Thus, we computed the CERES dependency score of the aggregated prognostic modules from the Achilles CRISPR-Cas9 screens of 975 cell lines (Meyers et al. 2017). Among the three primary aggregated prognostic modules (E2F targets, EMT, and inflammation), the E2F targets aggregated modules had significantly lower CERES scores (*P* < 0.01, 1000 permutations), indicating their essentiality for cell line survival. Conversely, the EMT and inflammation prognostic modules showed no cell line essentiality.

### Epigenetic regulation of network modules

**Figure 2.**
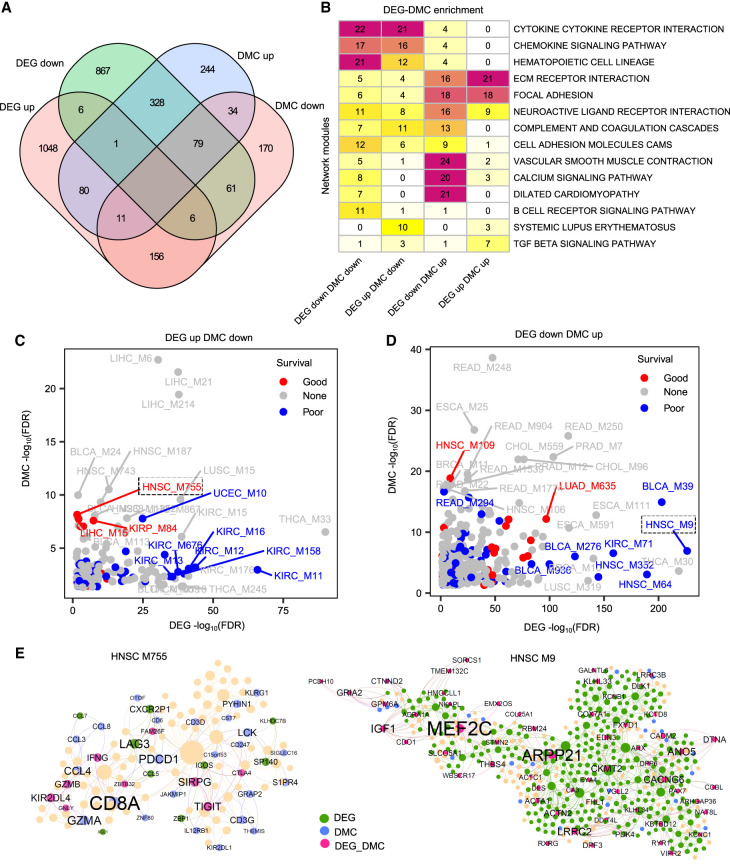
Regulation of module gene expression by DNA methylation. (*A*) Venn diagram showing the intersections between network modules enriched with different DEG and DMC categories in 16 cancer types. (*B*) Heatmap showing the top KEGG annotations for network modules with different DEG-DMC categories. The number on the heatmap corresponds to the frequency of module enrichment in each KEGG pathway. (*C*,*D*) Dot plots displaying the top 15 prognostic modules enriched for different DEG-DMC categories. The *x*-axis and *y*-axis indicate enrichment of DEGs and DMCs, respectively. (*E*) Two representative prognostic modules of HNSC. The network plots illustrate the two prognostic modules, M9 and M755, which are enriched for different DEG-DMC categories. In the network plot, the green, blue, and purple colors indicate DEGs, DMCs, and both DEG-DMCs, respectively. Node size is proportional to the connectivity of the network.

Epigenetic regulation through DNA methylation plays a crucial role in cancer development (Saghafinia et al. 2018; Locke et al. 2019). Therefore, we investigated the regulation of module-level DNA methylation and its impact on patient prognosis. We focused on 16 cancer types that had enough normal samples for comparative analysis with tumor samples. We identified 1517 and 1626 network modules significantly enriched for up-regulated and down-regulated differentially expressed genes (DEGs), respectively, whereas 849 and 597 network modules were enriched for up-regulated and down-regulated differentially methylated CpGs (DMCs), respectively. We observed a global inverse relationship between DEG- and DMC-enriched modules. For example, 408 modules were enriched for down-regulated DEGs and up-regulated DMCs (dDEGs-uDMCs), and 173 modules were enriched for up-regulated DEGs and down-regulated DMCs (uDEGs-dDMCs) (Fig. 2A). Modules enriched for dDEGs-uDMCs were related to vascular smooth muscle contraction and calcium signaling pathways, including the angiopoietin encoding gene *ANGPTL1* and the collagen encoding gene *COL14A1* (Fig. 2B). Meanwhile, network modules enriched for uDEGs-dDMCs were mostly involved in cytokine and chemokine pathways, suggesting coregulation of gene expression and methylation may play important roles in regulating different module functions.

Next, we analyzed the prognostic modules coregulated by both DEGs and DMCs using the pan-cancer networks. We conducted an intersection analysis of the survival-associated gene signature, DEG signature, and DMC-containing gene signature in the 16 cancer types. We found that 10,174 out of 37,353 (27%) survival-associated genes were also differentially expressed in these cancer types, and 15,478 out of 64,166 (24%) DEGs were also differentially methylated (Supplemental Fig. S3). Among the modules enriched with uDEGs-dDMCs, 13 (8%) and 34 (20%) modules were associated with good and poor patient survival, respectively (Fig. 2C). Similarly, among the dDEG-uDMC enriched modules, 34 (8%) and 65 (16%) modules were indicative of good and poor survivals, respectively (Fig. 2D). In head and neck squamous cell carcinoma (HNSC), module M755 was enriched for uDEG-dDMCs with good prognosis (hazard ratio (HR) = 0.63, *P* = 0.001) and mainly functions in natural killer (NK) cell–mediated cytotoxicity (aFET*P* = 1.7 × 10^−16^) (Fig. 2E). In contrast, module M9 of the HNSC network was enriched for dDEG-uDMCs with poor prognosis (HR = 1.4, *P* = 0.02) and mainly associated with the dilated cardiomyopathy pathway (aFET*P* = 3.5 × 10^−12^).

### Regulation of network modules by chromatin accessibility

**Figure 3.**
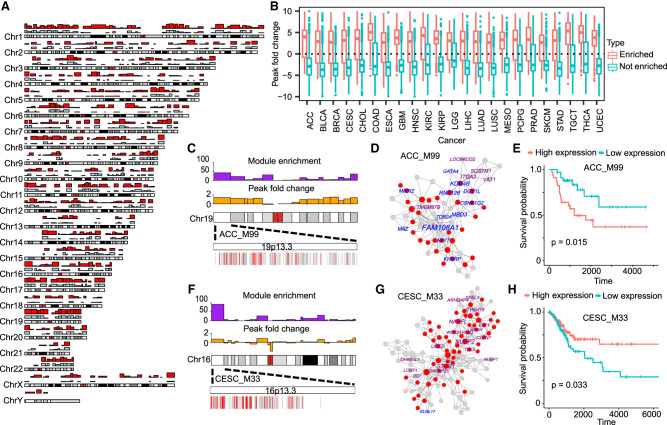
Regulation of network modules by chromatin accessibility. (*A*) Chromosomal cytobands with enriched network modules and open chromatins. Karyotype representation distinguishes euchromatin and heterochromatin regions using alternating light and dark shades, based on Giemsa banding. Bar plots in red and blue depict module-enriched regions and open chromatin regions, respectively. The *y*-axis represents the number of cancer types with the module enrichment and open chromatins, ranging from a minimum of zero to a maximum of 30. (*B*) Boxplot showing the fold changes of ATAC-seq peak signals in cytobands with enriched or depleted network modules compared with the genome background. (*C*) Prognostic module ACC_M99 enriched for the cytoband 19p13.3. The bar plots *above* the karyotype show module enrichment (purple) and ATAC-seq fold change (orange), respectively. The heatmap *below* the karyotype shows the enlarged cytoband region and the linear arrangement of module genes (red) in the cytoband. (*D*) Coexpression networks of ACC_M99. In the network plot, the red nodes indicate the genes in the 19p13.3 cytoband region. The names of prognostic genes are labeled by different colors (purple indicates good outcome; blue, bad outcome). (*E*) Survival plot of prognostic module ACC_M99. The high and low cutoffs were defined based on the median of gene expression across all tumor samples. (*F*–*H*) The cytoband enrichment (*F*), coexpression network (*G*), and survival plot (*H*) of prognostic module CESC_M33.

Given that chromatin configuration is a critical regulator of gene expression (Corces et al. 2018), our study investigated the regulation of network modules by chromatin accessibility in cancers. We examined whether network modules were enriched for specific chromosome locations that are influenced by chromatin states. The Giemsa banding technique stains euchromatic and heterochromatic regions with different GC contents, which generates a map of chromosome cytobands (Verma and Rees 1974). Thus, we used chromosome cytobands as the windows for the enrichment test. We found that 42% (11,568) of the network modules were significantly enriched in at least one cytoband of human chromosomes (gene overlaps > 3 and aFET*P* < 0.05), with 66% (568) of cytobands enriched in network modules of at least one cancer type. The cytobands with frequent module enrichment colocalized with open chromatin regions, as shown by the significantly higher ATAC-seq signals observed in module-enriched cytobands than in module-depleted cytobands (Wilcoxon test *P* < 2.2 × 10^−16^) (Fig. 3A,B). Using the Spearman's correlation test, we found that cytobands with a higher number of enriched modules tend to house more genes (r = 0.84, *P* = 9.8 × 10^−150^) (Supplemental Fig. S4), suggesting that coexpressed modules are inclined to reside within gene-rich regions of cytobands.

We also explored the enrichment of prognostic modules for chromosomal cytobands and found that 48% (414) of cytobands contained at least one prognostic module (Supplemental Table S5). Notably, 11 cytobands were enriched for prognostic modules in at least half of all cancer types analyzed, including three cytobands on Chromosome 19 (19p13.3, 19p13.11, and 19q13.43) (Supplemental Fig. S4). The cytoband 19p13.3 was enriched for prognostic modules in 17 cancers and contained half of the 60 genes in module M99 of adrenocortical carcinoma (ACC) (Fig. 3C–E). The ACC M99 eigengene was significantly associated with poor survival outcomes (HR = 2.7, *P* = 0.02). Seven prognostic genes of M99 were located on 19p13.3, including H3K79 methylation regulators *DOT1L* and *KDM4B*. The cytoband 16p13.3 was also enriched for prognostic modules of 22 cancer types. It contained 11 prognostic genes of M33 in cervical squamous cell carcinoma (CESC), including the cell-cycle repressor *E4F1* and the cytosolic iron–sulfur protein assembly component *CIAO3* (previously known as *NARFL*) (Fig. 3F–H). Both 19p13.3 and 16p13.3 were located in euchromatic regions with open chromatin status.

We used cell-type marker genes from the single-cell database PanglaoDB to explore whether cell-type marker genes were enriched in specific cytobands. Among the 862 cytobands in the human genome, 20 (2%) cytobands were enriched for cell-type marker genes (aFET*P* < 0.05, gene overlaps > 3). Similarly, among 11,568 network modules enriched in cytobands, 1265 (10%) modules were enriched for cell-type markers, suggesting that a small portion of cytobands/modules displays cell-type-specific expressions.

To investigate whether cytobands were also enriched for copy number variations, we analyzed the colocalization of module-enriched cytobands and the hotspots of copy number variations previously identified by the TCGA group using the GISTIC2.0 pipeline (Mermel et al. 2011). We found that seven of 11 cytobands with the most frequent enrichment of prognostic modules overlap with copy number variations in at least three cancer types (Supplemental Table S6). Some cytobands affected by copy number variations have been reported with crucial functions in cancer development. For example, 3p21.31 hosts a large number of tumor-suppressor genes and is frequently deleted in seven different cancer types (Jain et al. 2021). The chromosome region 1q21.3 has been reported with frequent amplifications, which can serve as a trackable biomarker and actionable target for breast cancer recurrence (Goh et al. 2017).

### The preservation of coexpressed modules across 32 cancers

**Figure 4.**
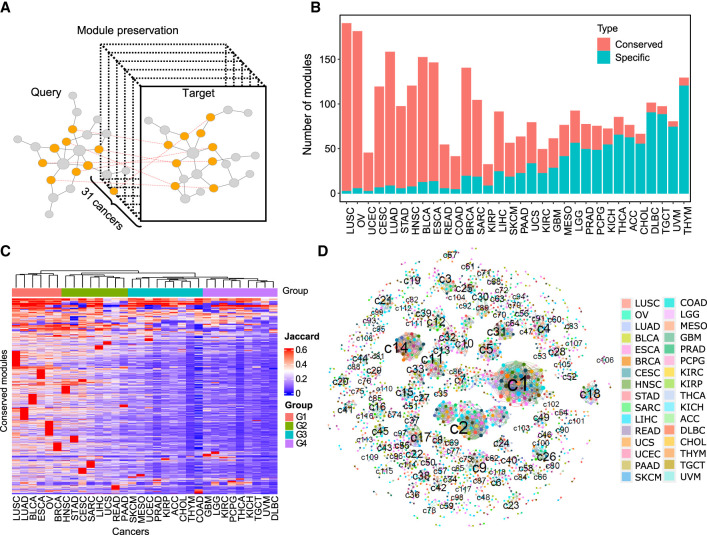
Preservation of pan-cancer network modules. (*A*) Schematic plot illustrating the module preservation analysis pipeline. (*B*) The number of conserved and cancer-specific modules identified in each cancer type. (*C*) Cancer-type clustering based on the similarity of conserved modules. The heatmap displays the conserved modules (n = 1941) as rows and the cancer types as columns. A hierarchical clustering algorithm was used to classify cancer types based on the Jaccard index of their conserved modules. (*D*) The module clusters formed by conserved modules. Each cluster represents a set of reciprocal conserved modules across different cancer types. The nodes represent conserved modules, and the colors indicate cancer types. The linked nodes indicate module clusters formed by conserved modules in different cancer types.

We investigated the preservation of network modules across 32 cancer types to reveal similarities in molecular regulatory mechanisms of cancer transcriptomes. To assess module preservation, we calculated the FET statistics and the Jaccard index of module similarity for pairwise module comparisons of all cancer types (Fig. 4A). We identified 829,167 module pairs with significant mutual similarity (aFET*P* < 0.05), of which 8020 module pairs (top 1%) were highly conserved with a Jaccard index > 0.4. These conserved module pairs came from 1941 modules (7% of total network modules) of 32 cancer types (Supplemental Table S7). In addition, we identified 1063 cancer-type-specific modules (4% of total network modules) that had little overlap with other cancer types (Jaccard index < 0.05 or aFET*P* > 0.05) (Supplemental Table S8). We selected these 1941 conserved modules and 1063 specific modules for detailed downstream analyses.

Each cancer contained a different number of conserved (six to 188) and specific (two to 120) modules. The ratio between the number of conserved and specific modules decreased from the highest in lung squamous cell carcinoma (LUSC) to the lowest in thymoma (THYM), which reflected the trend of shared transcriptomic signatures across all cancer types (Fig. 4B). We performed clustering analysis of 32 cancer types based on the Jaccard index of conserved modules, and classified the cancer types into four groups with different degrees of module similarity. The groups G1 and G2 consisted of 13 cancers with high similarity of conserved modules, whereas the groups G3 and G4 included 19 cancers with low module similarity (Fig. 4C). The ANOVA test revealed that the sample size was not significantly different in each cancer group (*P* > 0.1), suggesting sample size did not affect the module similarity during clustering analysis. Most cancers from G1 and G2 are from endoderm tissues and gynecologic tissues, consistent with previous observation that cancer types from anatomy-related tissues share high-molecular similarities (Hoadley et al. 2018).

### The prognostic features of conserved module clusters

We classified the 1941 conserved modules into 330 module clusters based on module similarities by a greedy algorithm (Supplemental Table S9). Each cluster corresponds to a set of conserved modules in multiple cancers, and 107 clusters contained reciprocal conserved modules in more than three cancer types (Fig. 4D). Gene Ontology (GO) annotation revealed that the conserved module clusters were related to functions such as chromatin assembly, homophilic cell adhesion, defense response to virus, and T and B cell activation pathways.

**Figure 5.**
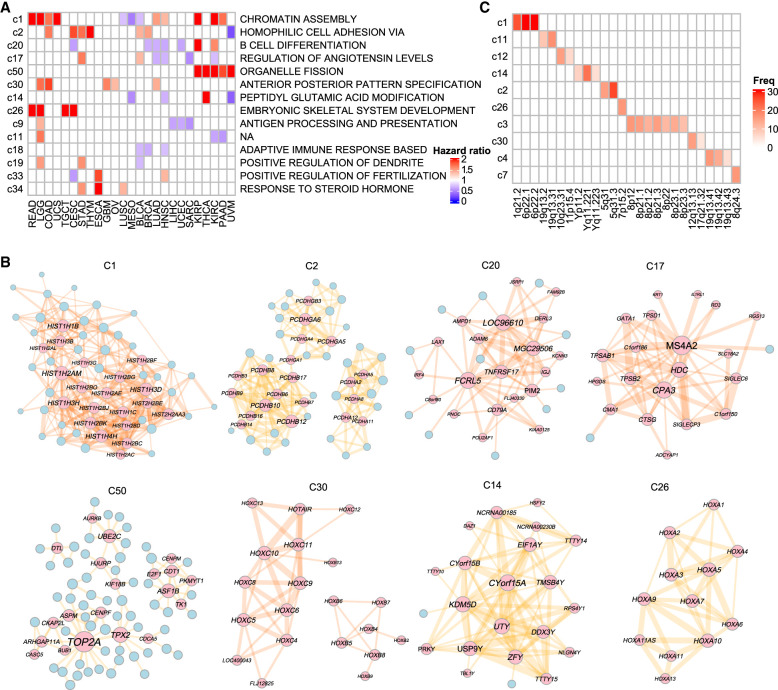
Characterization of conserved module clusters. (*A*) Prognostic significance and biological functions of pan-cancer conserved module clusters. The heatmap shows the hazard ratios of module clusters in at least three cancer types. The module clusters were annotated based on the most frequent GO process. (*B*) Aggregated networks of the top eight module clusters with the highest prognostic frequency in multiple cancers. The top 20 nodes with the highest connectivity are labeled. (*C*) Enrichment of module clusters in chromosomal cytobands. The heatmap illustrates the number of cancer types in which the module clusters show enrichment in the cytobands. The top 10 module clusters with the highest frequency of enrichments are shown.

Out of the 330 clusters, 97 (29%) were associated with patient survival in at least one cancer type, with 14 module clusters being associated with patient survival in at least three cancers. Notably, the cluster c1 eigengenes were associated with patient survival in 13 different cancer types, including 10 cancers with a high hazard ratio, indicating poor outcomes (Fig. 5A). The c1 module contained histone 1 family genes, which play essential roles in compacting chromatin and stabilizing high-order chromatin structures (Fig. 5B). The c2, c30, and c26 module clusters were primarily formed by closely related members of gene families such as *PCDHs* and *HOX* genes and were associated with a high hazard ratio in most cancer types. In contrast, module clusters c20 and c9, consisting mainly of immune genes, were associated with good outcomes. The modules in c20 regulated B cell development and function, whereas the c9 cluster contained *HLA* genes for antigen processing and presentation.

We observed that conserved module clusters had a tendency to localize in specific chromosomal regions. For instance, cluster c1 showed enrichment in 6p22.1 in 31 cancer types, whereas c2 was enriched in 5q31.3 in 26 cancer types (Fig. 5C). Among the 330 module clusters, 84 clusters were enriched for the same cytoband in more than three cancers. Furthermore, we found that the chromosome cytobands colocalizing with module clusters c1, c2, and c30, such as 6p22.1, 5q31.3, and 12q13.13, were also enriched for DEGs between tumor and normal samples (Supplemental Fig. S5). These findings suggest that conserved module clusters tend to be located in chromosomal hotspots that are frequently dysregulated in tumors.

### The prognostic modules unique to each cancer type

**Figure 6.**
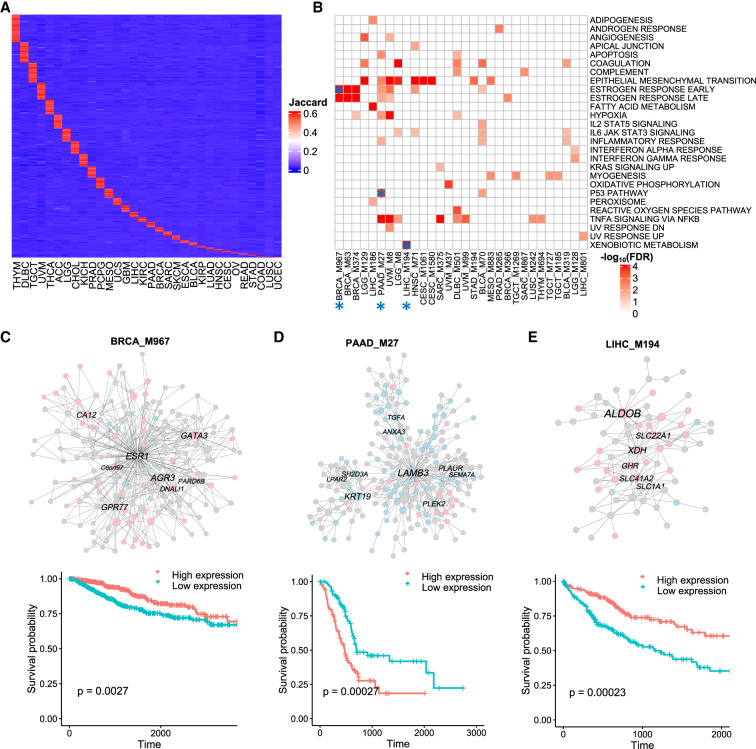
Cancer-type-specific prognostic modules. (*A*) Heatmap showing cancer-type-specific modules. Rows represent specific modules, and columns represent cancer types. Color intensity indicates the Jaccard index of the most similar module in each cancer type. (*B*) Heatmap showing the top 30 modules with significant enrichment for hallmark pathways. Color intensity in the heatmap is proportional to pathway enrichment. Asterisks highlight candidate cancer-specific modules for survival plots and unique biological functions. (*C*–*E*) Coexpression networks and survival plots of cancer-type-specific prognostic modules. In the network plots, the pink and light blue colors indicate genes associated with better and worse survivals, respectively. The names of hub genes in the network modules are labeled.

To further investigate the potential cancer-specific activities of network modules, we analyzed the cancer-type-specific modules across different cancer types (Fig. 6A). Among the 1063 cancer-type-specific modules identified, 252 (24%) were significantly associated with patient survival. Furthermore, 63 of these prognostic modules were significantly enriched for MSigDB hallmark pathways (Fig. 6B), indicating that these modules may play important roles in specific biological processes across different cancer types.

We observed that cancer-type-specific prognostic modules could have distinct functions in cancer development and signaling (Fig. 6C–E). For instance, breast cancer (BRCA) was associated with a module involved in estrogen response, consistent with the known role of estrogen in breast cancer development. Pancreatic adenocarcinoma (PAAD) was associated with a module related to the TP53 pathway, a key tumor-suppressor pathway that is frequently mutated in cancers. In liver cancer (LIHC), a unique module, M194, was related to xenobiotic metabolism, which is critical for detoxification of foreign substances and may contribute to the pathogenesis of liver cancer. Notably, among four modules unique to liver cancer, all of these cancer-specific modules were conserved in an independent non-TCGA cohort (Long et al. 2022). These results suggest that cancer-type-specific modules may have unique functions in cancer development and progression, highlighting their potential as cancer-type-specific regulatory mechanisms and therapeutic targets.

## Discussion

In this study, we performed a systematic investigation of coexpression networks across 32 cancer types. Compared with previous sporadic reports of cancer networks, our pan-cancer work represents a comprehensive analysis of all the major cancer types in the TCGA cohort using a uniform and well-established pipeline. We identified 4749 prognostic modules that play essential roles in cancer regulatory pathways, including cancer cell division, metastasis, and immune microenvironment processes. The network modules are regulated by multiscale mechanisms involving the interplay of gene expression, methylation, and chromatin accessibility. The network modules form preserved module clusters that preferentially reside in chromosomal hotspots. There are also cancer-type-specific prognostic modules that participate in cancer-specific biological processes. Together, our pan-cancer analysis provides a holistic view of the tumorigenesis of prognostic modules and revealed potential biomarkers for cancer development and progression.

It should be noted that TCGA transcriptomes were sequenced from bulk RNA-seq samples, and there were variations in the proportions of cell types in the sequenced samples that may confound individual gene or pathway analyses. However, the coexpression network approach is effective to partition genes from different cell types into distinct network modules, thereby capturing cell-type-specific modules that influence cancer prognosis. In a recent study of primary melanoma, we showed that the MEGENA coexpression network is an efficient tool for identifying modules of different cell types within the tumor microenvironment (Song et al. 2021). We have also shown the applicability of our network approach in identifying modules responsible for different cell types in bulk RNA-seq data sets from complex human diseases (Wang et al. 2019, 2021; Xu et al. 2022, 2023). Therefore, the network approach is able to unravel global connections of genes with related functions, minimize bias signals of survival prediction, and elucidate molecular regulations of cancer development.

Our analysis also revealed multiscale epigenetic regulations of network modules in different cancers. We observed that 42% of network modules were enriched for chromosome cytobands, which can be explained by epigenetic mechanisms for long-range regulations, such as chromatin accessibility and DNA methylation. Consistently, the module-enriched cytobands had higher ATAC-seq peak signals compared with the module-depleted cytobands, suggesting open chromatin regions may facilitate gene coexpression within the cytobands. DNA methylation is another important mechanism for long-range regulation. For example, the PCDH family genes from module cluster c2 have frequent methylation alterations in the multigene clusters of chromosome region 5q31 (Vega-Benedetti et al. 2019). Such methylation alterations reversely correlate with gene expressions in different cancers. We also observed that cytobands with prognostic module enrichment overlapped with copy number variations in multiple cancer types. In breast cancer, coexpressed genes tend to be connected at cytoband regions and coincide with known copy number altered regions (García-Cortés et al. 2020). These observations suggest copy number variation is an important player in regulating coexpression of network modules.

Finally, our results revealed the landscape of conserved and specific modules in different cancers. We found that the preserved signatures of network modules reflected the transcriptomic similarity of cancer types. Based on the similarity of conserved modules, cancers originating from anatomy-related tissues tended to cluster together. Consistently, a previous study reported similar patterns of cancer-type classification using multiomic integrative clustering from aneuploidy, CpG hypermethylation, mRNA, miRNA, and protein data sets (Hoadley et al. 2018). For example, the G1 and G2 groups with highly conserved network modules were primarily associated with gastrointestinal tumors (ESCA, READ, STAD), squamous histology cancers (LUSC, HNSC, CESC, ESCA, BLCA), and pangyn cancers (BRCA, OV, CESC, UCS). This suggests that cancer-type clustering is primarily organized by histology and tissue type, and module-based analysis can be potentially served as an approach for cancer-type classification.

In summary, our study provides a comprehensive understanding of the pan-cancer coexpression landscape and identifies prognostic modules that play crucial roles in cancer regulatory pathways. The multiscale regulations of prognostic modules, including their coregulation at the chromosome level and their associations with DNA methylation, offer new insights into the underlying mechanisms of cancer development and progression. We also shared the networks, modules, prognostic features, and their functional annotations on open-source websites, allowing future work to use, evaluate, and explore a wide range of cancer biology questions. Our comprehensive pan-cancer results offer a unique opportunity for comparative network analysis in this field.

## Methods

### Clinical and molecular data sets

We obtained standardized, normalized, and batch-corrected data matrices from the Pan-Cancer Atlas of TCGA. Clinical matrices of patients and samples, normalized RNA-seq data sets, and processed DNA methylation values from Illumina 450K BeadChip arrays were downloaded from the publication page (https://gdc.cancer.gov/about-data/publications/pancanatlas). Gene expression data sets were generated using the Firehose pipeline with MapSplice and RSEM (Wang et al. 2010; Li and Dewey 2011), and were normalized by setting the upper-quartile to 1000. It also corrected for batch effects, such as platform variations of HiSeq and GA, as well as batch IDs in certain cancer types, based on RNASeqV2 mRNA data (Synapse syn4976363). Normalized ATAC-seq peak calls were downloaded from the website (Corces et al. 2018; https://gdc.cancer.gov/about-data/publications/ATACseq-AWG). We used overall survival and progression-free intervals from the curated clinical data resource (Liu et al. 2018), as recommended by the Pan-Cancer Atlas, as clinical endpoints for survival outcome analysis. We distinguished tumor and normal samples by the sample type code “01A” (primary solid tumor) and “11A” (solid tissue normal), respectively. We only used solid tumors with more than 50 samples for network analyses.

### Coexpression network construction

The normalized, batch-corrected, and platform-corrected RNA-seq data sets were divided into 32 matrices based on cancer types. We filtered out lowly expressed genes that contained NA values or zero values in >75% of samples for each cancer and log_2_-transformed gene expressions. As confounding factors like batches, gender, and age can systematically influence gene expression levels and correlations during coexpression network analysis, we adjusted for confounding factors such as race, age, and gender using a linear model (Song et al. 2021; Xu et al. 2023). In this model, we fitted these confounding factors and captured the residuals using the lm() function from the R software (v3.6) (R Core Team 2018).

Coexpression networks were built by MEGENA for each cancer type, following our previous pipelines (Song and Zhang 2015; Wang et al. 2019, 2021; Song et al. 2021). We permuted (n = 10) the gene expression matrix across the samples to calculate the false-positive rate (FPR) and the corresponding false-discovery rate (FDR) for each correlation coefficient cutoff. Then an FDR threshold of 0.05 was applied to determine the correlation coefficient cutoff that effectively filtered out nonsignificant correlations. The gene pairs of significance were initially sorted by absolute Pearson correlation coefficients. Sequentially, the sorted gene pairs were examined if each pair can be placed on the three-dimensional topological sphere without intersecting with other edges, a process referred to as the planarity test. The resulting coexpression network formed part of a category of geometrical networks known as planar filtered networks (PFNs), which can be depicted on a sphere's surface without any link intersections (Tumminello et al. 2005). Subsequently, the PFN underwent an unsupervised clustering process to identify network clusters (e.g., gene modules) at various compactness resolutions through multiscale clustering analysis (MCA). MCA divided the parent module into child modules by searching for an optimal partition for Newman's modularity (Q) (Newman 2006). Consequently, the resulting gene modules were structured in a hierarchy that represents a multiscale organization of gene modules with varying compactness degrees. We executed multiscale hub analysis by pinpointing nodes with significantly (*P* < 0.05) higher network connectivity than the randomly permuted planar networks.

### Module enrichment and prognosis analysis

We used ClusterProfiler for enrichment testing of coexpressed modules (Yu et al. 2012). To annotate the pathway functions of network modules, we performed FET to determine whether the module genes were enriched for GO processes, KEGG, REACTOME, and hallmark pathways from MSigDB. We adjusted the multitesting *P*-values using the Benjamini–Hochberg (BH) method. To test the cytoband enrichment, we downloaded gene symbols and cytoband locations of the GRCh38/hg38 reference from the HGNC database. We applied FET from ClusterProfiler to test whether genes from each cytoband were enriched for a network module. We adjusted the multitesting *P*-values using the BH method. We defined the significant cytoband enrichment with a cutoff of overlapping size greater than three and aFET*P* < 0.05.

We obtained the clinical endpoints from the curated clinical data resource for module prognosis analysis (Liu et al. 2018). In accordance with a previous study, overall survival was used as an endpoint in 24 cancer types, whereas progression-free intervals were used in nine cancer types with few deaths, including BRCA, DLBC, LGG, PCPG, PRAD, READ, TGCT, THCA, and THYM (Smith and Sheltzer 2022). Next, we used PCA to calculate the module eigengenes, which represent the first PC of gene expressions. We stratified module eigengenes into high- and low-expression groups based on their relative expressions compared with the median level. To test whether expressions of module eigengenes significantly affect survival time (*P* < 0.05), we built a Cox proportional-hazard model using the R function “coxph.” The hazard ratio reflects how the expression levels influence the rate of patient survival, in which an increase in the death hazard results in a decrease in the length of survival. Similarly, we identified prognostic genes in each cancer by fitting their covariate-adjusted expressions to the Cox proportional-hazard model.

### DEG and DMC analysis

We performed DEG analysis by comparing normalized gene expressions between tumor and normal tissues in 17 cancer types. We performed differential expression analysis using the moderated *t*-test implemented in the limma package (Ritchie et al. 2015). Patient age, gender, and race were treated as covariates in the design matrix. We filtered significant DEGs with a fold change greater than two and adjusted *P*-value < 0.05.

For DMC analysis, we identified gene promoters within 2 kb upstream of and 200 bp downstream from the transcription start sites using the R function “promoters” from the “ensembldb” package. We converted the CpG locations of Illumina 450 K BeadChip Arrays to hg38 coordinates using the R function “liftOver” and compared them with the promoter regions using the “findOverlaps” function from the “GenomicRanges” package. We used resulting promoter-located CpG probes to identify DMCs between tumor and normal samples in 16 cancer types. Similar to DEG analysis, we analyzed the beta values of CpG probes by the moderated *t*-test implemented in the limma package, with the adjustment of age, gender, and race as covariates (Ritchie et al. 2015). We further filtered significant DMCs by a beta value change > 0.2 and adjusted *P*-value < 0.05. We filtered out CpG probes that were potentially affected by SNPs if the containing SNPs had a minor allele frequency higher than 0.05.

### ATAC-seq peak signal analysis

We confined our analysis to tumor samples from the ATAC-seq data sets in each cancer type. We used the normalized and log_2_-transformed pan-cancer peak set counts from the Pan-Cancer Atlas for ATAC-seq analysis (Corces et al. 2018). For each fixed-width genome region, we used “fixed-width peaks” and normalized the peak values across all samples within each cancer type. These “fixed-width peaks” facilitate direct comparisons of ATAC peak values among different samples. We determined the peak value of each cytoband by taking the average of the peak counts of the fixed-width regions within that cytoband. The R function “subsetByOverlaps” was used to compare the chromosomal coordinates between the cytoband and ATAC-seq peak sets. We calculated the genome background signals by averaging all peak set values from all the samples. The fold change of the cytobands was calculated by dividing the mean count values of the peak sets by that of the genome background. The Wilcoxon rank test was applied to test the significance of the fold change.

### Module preservation analysis

We performed module preservation analysis by calculating module similarities in all cancer types. We compared the module genes of a cancer type with the network modules in other cancer types and calculated pairwise module similarity using the Jaccard index, where A and B indicate the genes from each module:

$$J(A,\;B)=\frac{\left|A\;\cap \;B\right|}{\left|A\;\cup \;B\right|}.$$

We then applied the FET of ClusterProfiler to calculate the significance of module similarity in each cancer type and used the BH method to adjust the multitesting *P*-values (Yu et al. 2012). We defined conserved modules as those with a Jaccard index > 0.4 and aFET*P* < 0.05 and defined specific modules as those with a Jaccard index < 0.05 or aFET*P* > 0.05. We used module sizes with 10 to 500 genes for the enrichment test of the preservation analysis, as suggested by ClusterProfiler.

As conserved modules have their counterparts in different cancer types, we grouped them into module clusters using the greedy algorithm. The algorithm implemented a loop to iteratively search module clusters, which included three steps: (1) starting from a random module, the algorithm identified the conserved counterparts of the target module; (2) the identified modules were pooled together to search for more counterparts until all the conserved modules were identified; and (3) the identified conserved modules were grouped into one module cluster and excluded from the search list. We repeated the steps until all conserved modules were assigned to module clusters.

### Aggregation of network modules

We performed network aggregation in the pan-cancer study to illustrate the consensus prognostic modules or conserved modules. First, we extracted the genes of the target modules to obtain the nodes of aggregated networks for one cancer type. Next, we used the resulting nodes to search the MEGENA network to obtain their coexpression relationships and generate the network edges. We merged the network nodes and edges from different cancer types to construct the aggregated network (Xu et al. 2022). We calculated the conservation weights of network nodes based on the total frequency of the node genes present in different cancer types. Similarly, we calculated the conservation weights of network edges based on the total frequency of the gene–gene coexpression links in different cancer types. To obtain a global coexpression network conserved in more than three cancer types, we filtered the less conserved nodes with weights less than four and generated the final aggregated network by merging the edges from the remaining conserved nodes.

## Data access

The summarized files of MEGENA coexpression networks and prognostic modules in 32 cancer types can be found in Supplemental Tables S1 and S2, respectively. The code for network analysis is available in the Supplemental Code. The comprehensive results of networks and modules in each cancer type, along with coding scripts, are available at GitHub (https://github.com/penguab/Pancancer_Networks), Zenodo (https://doi.org/10.5281/zenodo.8271601), and as Supplemental File S1.

## Supplementary Material

Supplement 1

Supplement 2

Supplement 3

Supplement 4

Supplement 5

Supplement 6

Supplement 7

Supplement 8

Supplement 9

Supplement 10

Supplement 11

Supplement 12

Supplement 13

Supplement 14

Supplement 15

Supplement 16

## References

[GR278063XUC1] Chella Krishnan K, Kurt Z, Barrere-Cain R, Sabir S, Das A, Floyd R, Vergnes L, Zhao Y, Che N, Charugundla S, 2018. Integration of multi-omics data from mouse diversity panel highlights mitochondrial dysfunction in non-alcoholic fatty liver disease. Cell Syst 6: 103–115.e7. 10.1016/j.cels.2017.12.006 29361464 PMC5799036

[GR278063XUC2] Corces MR, Granja JM, Shams S, Louie BH, Seoane JA, Zhou W, Silva TC, Groeneveld C, Wong CK, Cho SW, 2018. The chromatin accessibility landscape of primary human cancers. Science 362: eaav1898. 10.1126/science.aav1898 30361341 PMC6408149

[GR278063XUC3] Creixell P, Reimand J, Haider S, Wu G, Shibata T, Vazquez M, Mustonen V, Gonzalez-Perez A, Pearson J, Sander C, 2015. Pathway and network analysis of cancer genomes. Nat Methods 12: 615–621. 10.1038/nmeth.3440 26125594 PMC4717906

[GR278063XUC4] García-Cortés D, de Anda-Jáuregui G, Fresno C, Hernández-Lemus E, Espinal-Enríquez J. 2020. Gene co-expression is distance-dependent in breast cancer. Front Oncol 10: 1232. 10.3389/fonc.2020.01232 32850369 PMC7396632

[GR278063XUC5] Goh JY, Feng M, Wang W, Oguz G, Yatim SMJM, Lee PL, Bao Y, Lim TH, Wang P, Tam WL, 2017. Chromosome 1q21.3 amplification is a trackable biomarker and actionable target for breast cancer recurrence. Nat Med 23: 1319–1330. 10.1038/nm.4405 28967919

[GR278063XUC6] Hanahan D, Weinberg RA. 2011. Hallmarks of cancer: the next generation. Cell 144: 646–674. 10.1016/j.cell.2011.02.013 21376230

[GR278063XUC7] Hoadley KA, Yau C, Hinoue T, Wolf DM, Lazar AJ, Drill E, Shen R, Taylor AM, Cherniack AD, Thorsson V, 2018. Cell-of-origin patterns dominate the molecular classification of 10,000 tumors from 33 types of cancer. Cell 173: 291–304.e6. 10.1016/j.cell.2018.03.022 29625048 PMC5957518

[GR278063XUC8] Jain Y, Chandradoss KR, A VA, Bhattacharya J, Lal M, Bagadia M, Singh H, Sandhu KS. 2021. Convergent evolution of a genomic rearrangement may explain cancer resistance in hystrico- and sciuromorpha rodents. NPJ Aging Mech Dis 7: 20. 10.1038/s41514-021-00072-9 34471123 PMC8410860

[GR278063XUC9] Langfelder P, Horvath S. 2008. WGCNA: an R package for weighted correlation network analysis. BMC Bioinformatics 9: 559. 10.1186/1471-2105-9-559 19114008 PMC2631488

[GR278063XUC10] Li B, Dewey CN. 2011. RSEM: accurate transcript quantification from RNA-seq data with or without a reference genome. BMC Bioinformatics 12: 323. 10.1186/1471-2105-12-323 21816040 PMC3163565

[GR278063XUC11] Liu J, Lichtenberg T, Hoadley KA, Poisson LM, Lazar AJ, Cherniack AD, Kovatich AJ, Benz CC, Levine DA, Lee AV, 2018. An integrated TCGA pan-cancer clinical data resource to drive high-quality survival outcome analytics. Cell 173: 400–416.e11. 10.1016/j.cell.2018.02.052 29625055 PMC6066282

[GR278063XUC12] Locke WJ, Guanzon D, Ma C, Liew YJ, Duesing KR, Fung KYC, Ross JP. 2019. DNA methylation cancer biomarkers: translation to the clinic. Front Genet 10: 1150. 10.3389/fgene.2019.01150 31803237 PMC6870840

[GR278063XUC13] Long M, Zhou Z, Wei X, Lin Q, Qiu M, Zhou Y, Chen P, Jiang Y, Wen Q, Liu Y, 2022. A novel risk score based on immune-related genes for hepatocellular carcinoma as a reliable prognostic biomarker and correlated with immune infiltration. Front Immunol 13: 1023349. 10.3389/fimmu.2022.1023349 36353638 PMC9637590

[GR278063XUC14] Mermel CH, Schumacher SE, Hill B, Meyerson ML, Beroukhim R, Getz G. 2011. GISTIC2.0 facilitates sensitive and confident localization of the targets of focal somatic copy-number alteration in human cancers. Genome Biol 12: R41. 10.1186/gb-2011-12-4-r41 21527027 PMC3218867

[GR278063XUC15] Meyers RM, Bryan JG, McFarland JM, Weir BA, Sizemore AE, Xu H, Dharia NV, Montgomery PG, Cowley GS, Pantel S, 2017. Computational correction of copy number effect improves specificity of CRISPR-Cas9 essentiality screens in cancer cells. Nat Genet 49: 1779–1784. 10.1038/ng.3984 29083409 PMC5709193

[GR278063XUC16] Newman ME. 2006. Modularity and community structure in networks. Proc Natl Acad Sci 103: 8577–8582. 10.1073/pnas.0601602103 16723398 PMC1482622

[GR278063XUC17] Raina P, Guinea R, Chatsirisupachai K, Lopes I, Farooq Z, Guinea C, Solyom CA, de Magalhães JP. 2023. GeneFriends: gene co-expression databases and tools for humans and model organisms. Nucleic Acids Res 51: D145–D158. 10.1093/nar/gkac1031 36454018 PMC9825523

[GR278063XUC18] R Core Team. 2018. R: a language and environment for statistical computing. R Foundation for Statistical Computing, Vienna. https://www.R-project.org/.

[GR278063XUC19] Ritchie ME, Phipson B, Wu D, Hu Y, Law CW, Shi W, Smyth GK. 2015. *limma* powers differential expression analyses for RNA-sequencing and microarray studies. Nucleic Acids Res 43: e47. 10.1093/nar/gkv007 25605792 PMC4402510

[GR278063XUC20] Rooney MS, Shukla SA, Wu CJ, Getz G, Hacohen N. 2015. Molecular and genetic properties of tumors associated with local immune cytolytic activity. Cell 160: 48–61. 10.1016/j.cell.2014.12.033 25594174 PMC4856474

[GR278063XUC21] Saghafinia S, Mina M, Riggi N, Hanahan D, Ciriello G. 2018. Pan-cancer landscape of aberrant DNA methylation across human tumors. Cell Rep 25: 1066–1080.e8. 10.1016/j.celrep.2018.09.082 30355485

[GR278063XUC22] Smith JC, Sheltzer JM. 2022. Genome-wide identification and analysis of prognostic features in human cancers. Cell Rep 38: 110569. 10.1016/j.celrep.2022.110569 35354049 PMC9042322

[GR278063XUC23] Song WM, Zhang B. 2015. Multiscale embedded gene co-expression network analysis. PLoS Comput Biol 11: e1004574. 10.1371/journal.pcbi.1004574 26618778 PMC4664553

[GR278063XUC24] Song WM, Agrawal P, Von Itter R, Fontanals-Cirera B, Wang M, Zhou X, Mahal LK, Hernando E, Zhang B. 2021. Network models of primary melanoma microenvironments identify key melanoma regulators underlying prognosis. Nat Commun 12: 1214. 10.1038/s41467-021-21457-0 33619278 PMC7900178

[GR278063XUC25] Tumminello M, Aste T, Di Matteo T, Mantegna RN. 2005. A tool for filtering information in complex systems. Proc Natl Acad Sci 102: 10421–10426. 10.1073/pnas.0500298102 16027373 PMC1180754

[GR278063XUC26] Vega-Benedetti AF, Loi E, Moi L, Blois S, Fadda A, Antonelli M, Arcella A, Badiali M, Giangaspero F, Morra I, 2019. Clustered protocadherins methylation alterations in cancer. Clin Epigenetics 11: 100. 10.1186/s13148-019-0695-0 31288858 PMC6617643

[GR278063XUC27] Verma SC, Rees H. 1974. Giemsa staining and the distribution of heterochromatin in rye chromosomes. Heredity (Edinb) 32: 118–122. 10.1038/hdy.1974.12

[GR278063XUC28] Wang K, Singh D, Zeng Z, Coleman SJ, Huang Y, Savich GL, He X, Mieczkowski P, Grimm SA, Perou CM, 2010. MapSplice: accurate mapping of RNA-seq reads for splice junction discovery. Nucleic Acids Res 38: e178. 10.1093/nar/gkq622 20802226 PMC2952873

[GR278063XUC29] Wang Q, Zhang Y, Wang M, Song WM, Shen Q, McKenzie A, Choi I, Zhou X, Pan PY, Yue Z, 2019. The landscape of multiscale transcriptomic networks and key regulators in Parkinson's disease. Nat Commun 10: 5234. 10.1038/s41467-019-13144-y 31748532 PMC6868244

[GR278063XUC31] Wang M, Li A, Sekiya M, Beckmann ND, Quan X, Schrode N, Fernando MB, Yu A, Zhu L, Cao J, 2021. Transformative network modeling of multi-omics data reveals detailed circuits, key regulators, and potential therapeutics for Alzheimer's disease. Neuron 109: 257–272.e14. 10.1016/j.neuron.2020.11.002 33238137 PMC7855384

[GR278063XUC32] Xu P, Wang M, Song WM, Wang Q, Yuan GC, Sudmant PH, Zare H, Tu Z, Orr ME, Zhang B. 2022. The landscape of human tissue and cell type specific expression and co-regulation of senescence genes. Mol Neurodegener 17: 5. 10.1186/s13024-021-00507-7 35000600 PMC8744330

[GR278063XUC33] Xu P, Wang M, Sharma NK, Comeau ME, Wabitsch M, Langefeld CD, Civelek M, Zhang B, Das SK. 2023. Multi-omic integration reveals cell-type-specific regulatory networks of insulin resistance in distinct ancestry populations. Cell Syst 14: 41–57.e8. 10.1016/j.cels.2022.12.005 36630956 PMC9852073

[GR278063XUC34] Yang Y, Han L, Yuan Y, Li J, Hei N, Liang H. 2014. Gene co-expression network analysis reveals common system-level properties of prognostic genes across cancer types. Nat Commun 5: 3231. 10.1038/ncomms4231 24488081 PMC3951205

[GR278063XUC35] Yu G, Wang LG, Han Y, He QY. 2012. clusterProfiler: an R package for comparing biological themes among gene clusters. OMICS 16: 284–287. 10.1089/omi.2011.0118 22455463 PMC3339379

[GR278063XUC36] Yu LH, Huang QW, Zhou XH. 2019. Identification of cancer hallmarks based on the gene co-expression networks of seven cancers. Front Genet 10: 99. 10.3389/fgene.2019.00099 30838028 PMC6389798

[GR278063XUC37] Zhang B, Horvath S. 2005. A general framework for weighted gene co-expression network analysis. Stat Appl Genet Mol Biol 4: Article17. 10.2202/1544-6115.1128 16646834

