# Supplementary material for: Multiscale network modeling reveals the gene regulatory landscape driving cancer prognosis in 32 cancer types
Source: Genome Res. 2023 Oct;33(10):1806–17. doi: 10.1101/gr.278063.123 (PMC10691533; doi:10.1101/gr.278063.123)
Supplement: Supplement 1 [file Supplemental_Fig_S1.docx]

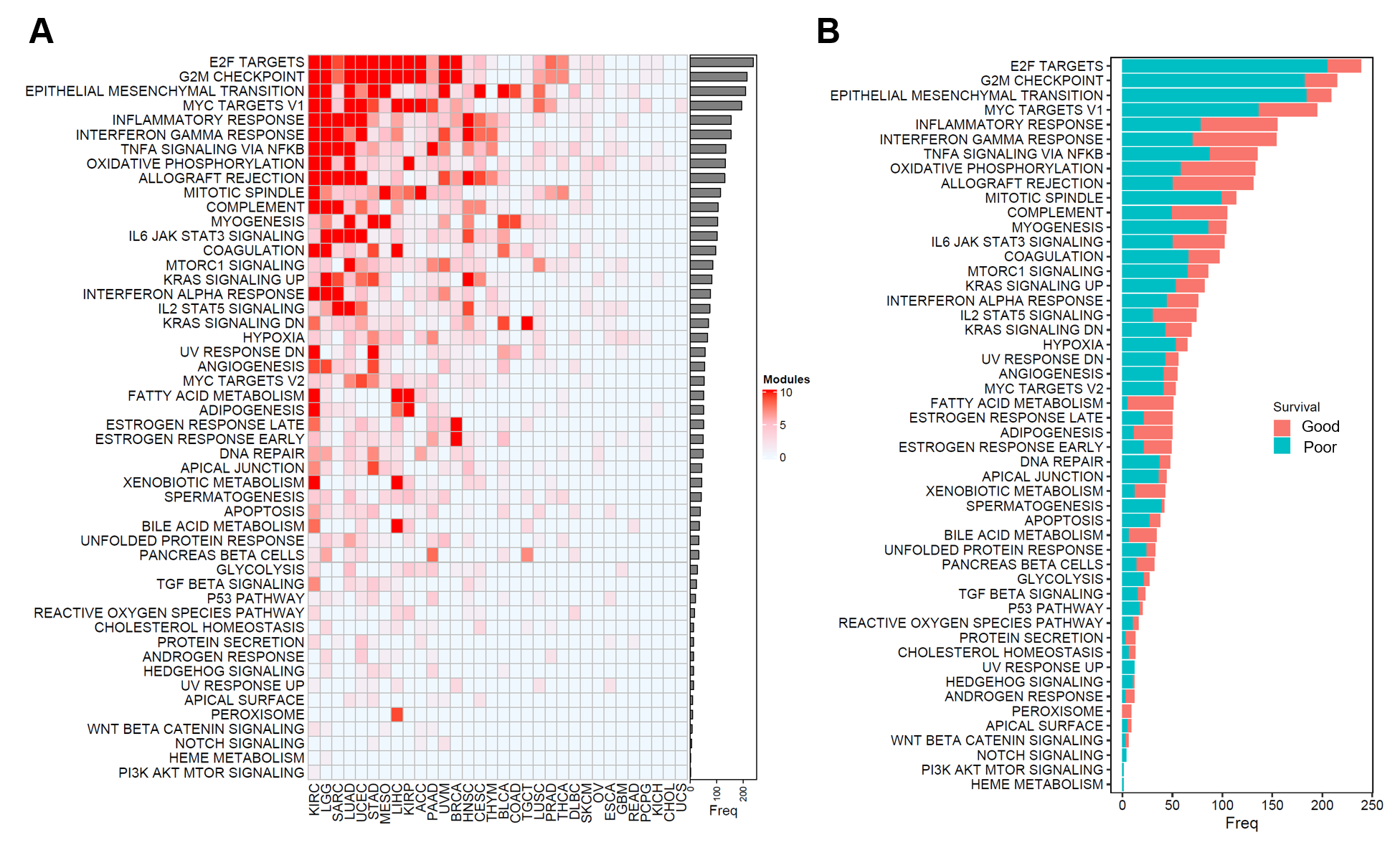


**Supplemental Fig. S1 The biological functions of the prognostic modules in 32 cancer types. A)** Heatmap showing the number of prognostic modules enriched for the 50 MsigDB hallmark pathways in each cancer type. The color intensity is proportional to the number of enriched modules. **B)** Bar plot showing the number of prognostic modules associated with different survival outcomes in each MsigDB hallmark pathway. The colors indicate different types of survival outcomes (pink: good outcome; green: poor outcome).
