# Supplementary material for: Multiscale network modeling reveals the gene regulatory landscape driving cancer prognosis in 32 cancer types
Source: Genome Res. 2023 Oct;33(10):1806–17. doi: 10.1101/gr.278063.123 (PMC10691533; doi:10.1101/gr.278063.123)
Supplement: Supplement 2 [file Supplemental_Fig_S2.docx]

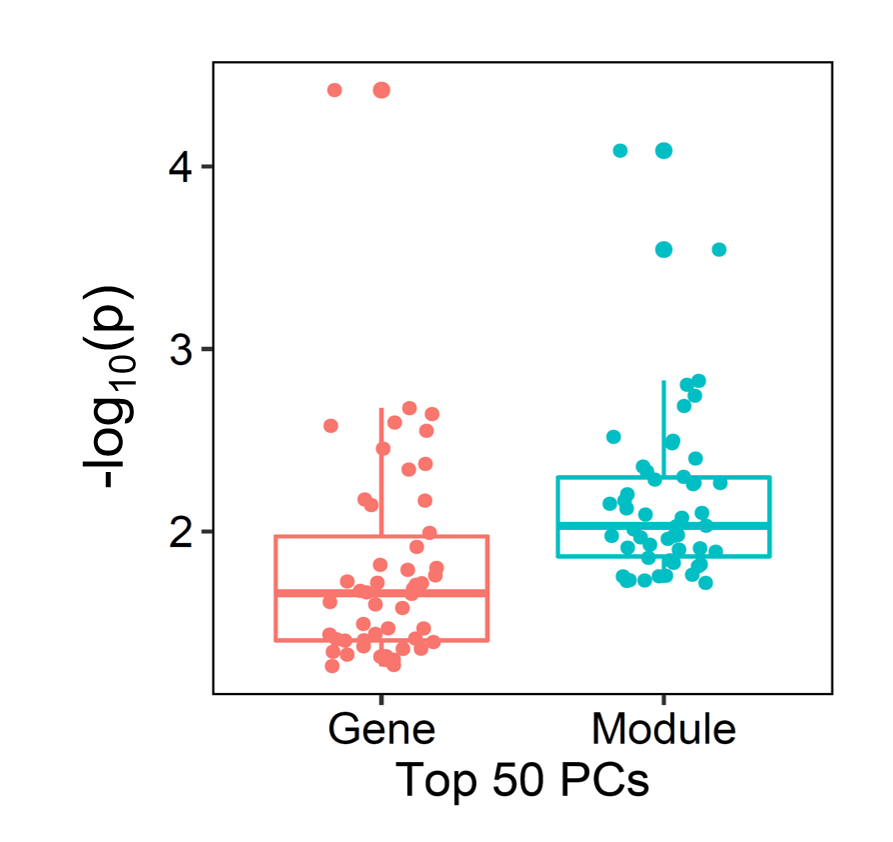


**Supplemental Fig. S2 Box plot showing prediction significance of top 50 PCs of prognostic genes and prognostic modules.** Each dot indicates a PC of prognostic genes and network modules. The Y axis shows the significance of the PC in survival prediction, with the p-value calculated from the Cox proportional-hazards model.
