# Supplementary material for: Multiscale network modeling reveals the gene regulatory landscape driving cancer prognosis in 32 cancer types
Source: Genome Res. 2023 Oct;33(10):1806–17. doi: 10.1101/gr.278063.123 (PMC10691533; doi:10.1101/gr.278063.123)
Supplement: Supplement 3 [file Supplemental_Fig_S3.docx]

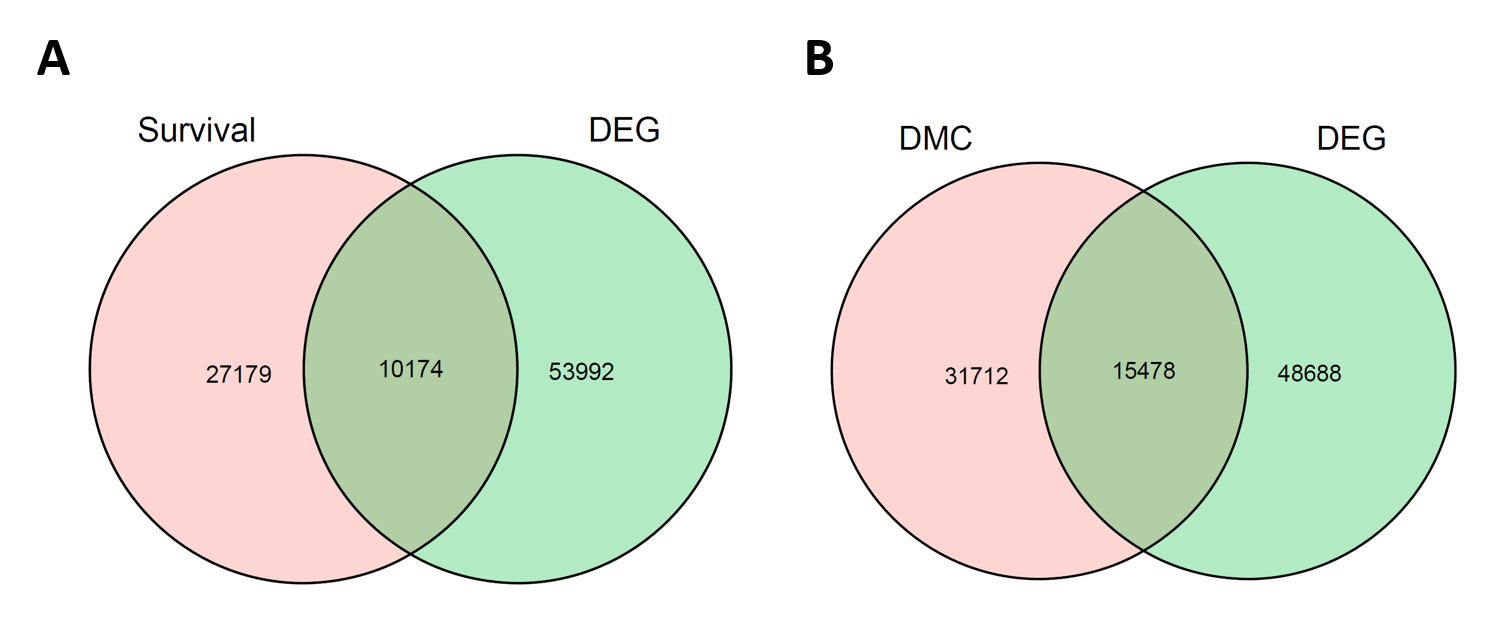


**Fig. S3 Venn plot showing the intersections of genes from different categories. A)** The intersection of genes associated with survival and differential expression (DEG). **B)** The intersection of genes with differential expression (DEG) and differential methylation (DMC).
