# Supplementary material for: Multiscale network modeling reveals the gene regulatory landscape driving cancer prognosis in 32 cancer types
Source: Genome Res. 2023 Oct;33(10):1806–17. doi: 10.1101/gr.278063.123 (PMC10691533; doi:10.1101/gr.278063.123)
Supplement: Supplement 4 [file Supplemental_Fig_S4.docx]

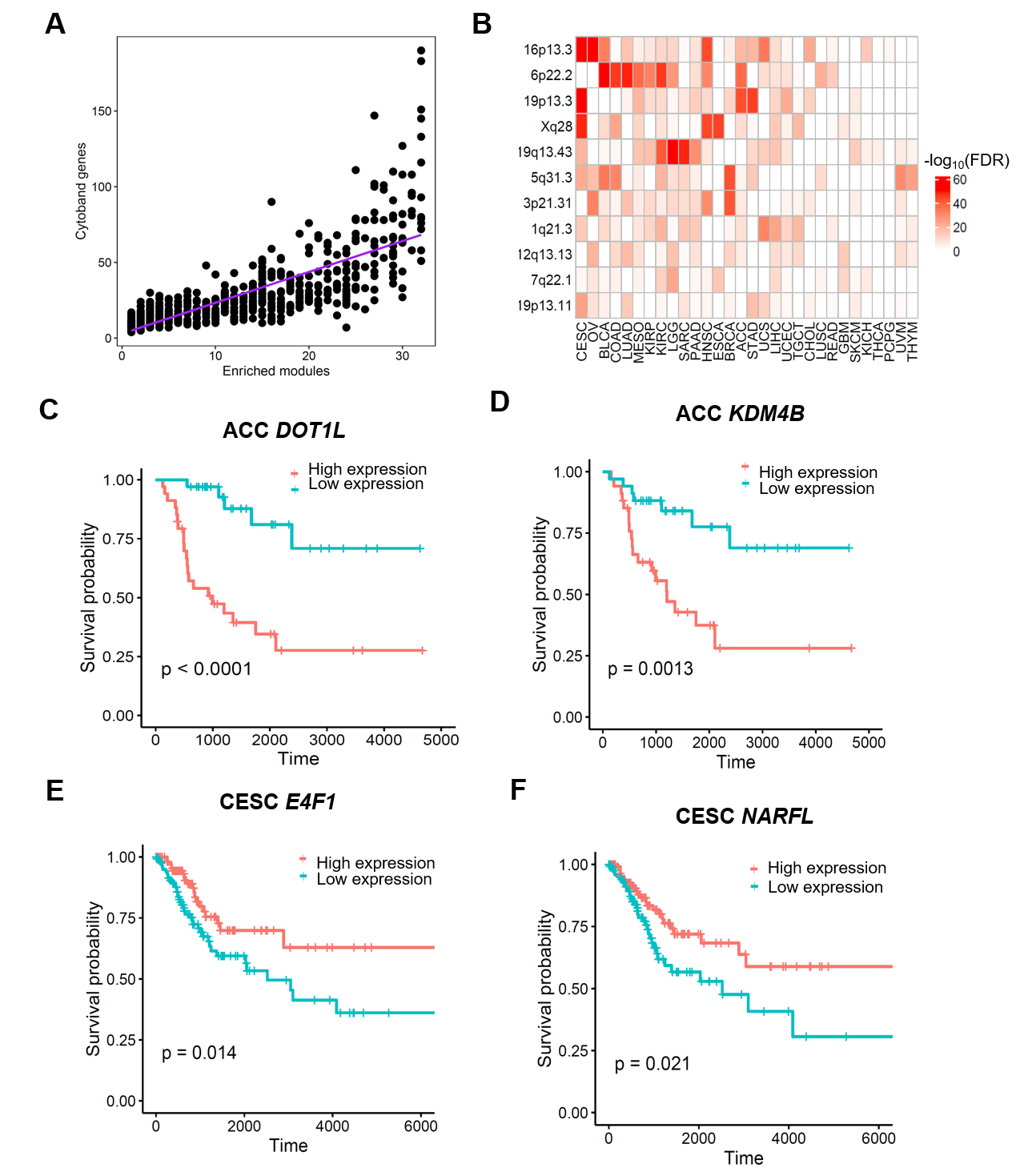


**Supplemental Fig. S4 The cytobands enriched for prognostic modules. A)** Dot plot showing correlation of enriched module frequency with the number of containing genes in chromosome cytobands. **B)** The enrichment of prognostic modules in cytobands across more than 16 cancer types. The heatmap displays the color intensity in proportion to the significance of enrichment for prognostic modules. **C-F)** Survival plots of candidate genes in the cytoband-enriched modules.
