# Supplementary material for: Multiscale network modeling reveals the gene regulatory landscape driving cancer prognosis in 32 cancer types
Source: Genome Res. 2023 Oct;33(10):1806–17. doi: 10.1101/gr.278063.123 (PMC10691533; doi:10.1101/gr.278063.123)
Supplement: Supplement 5 [file Supplemental_Fig_S5.docx]

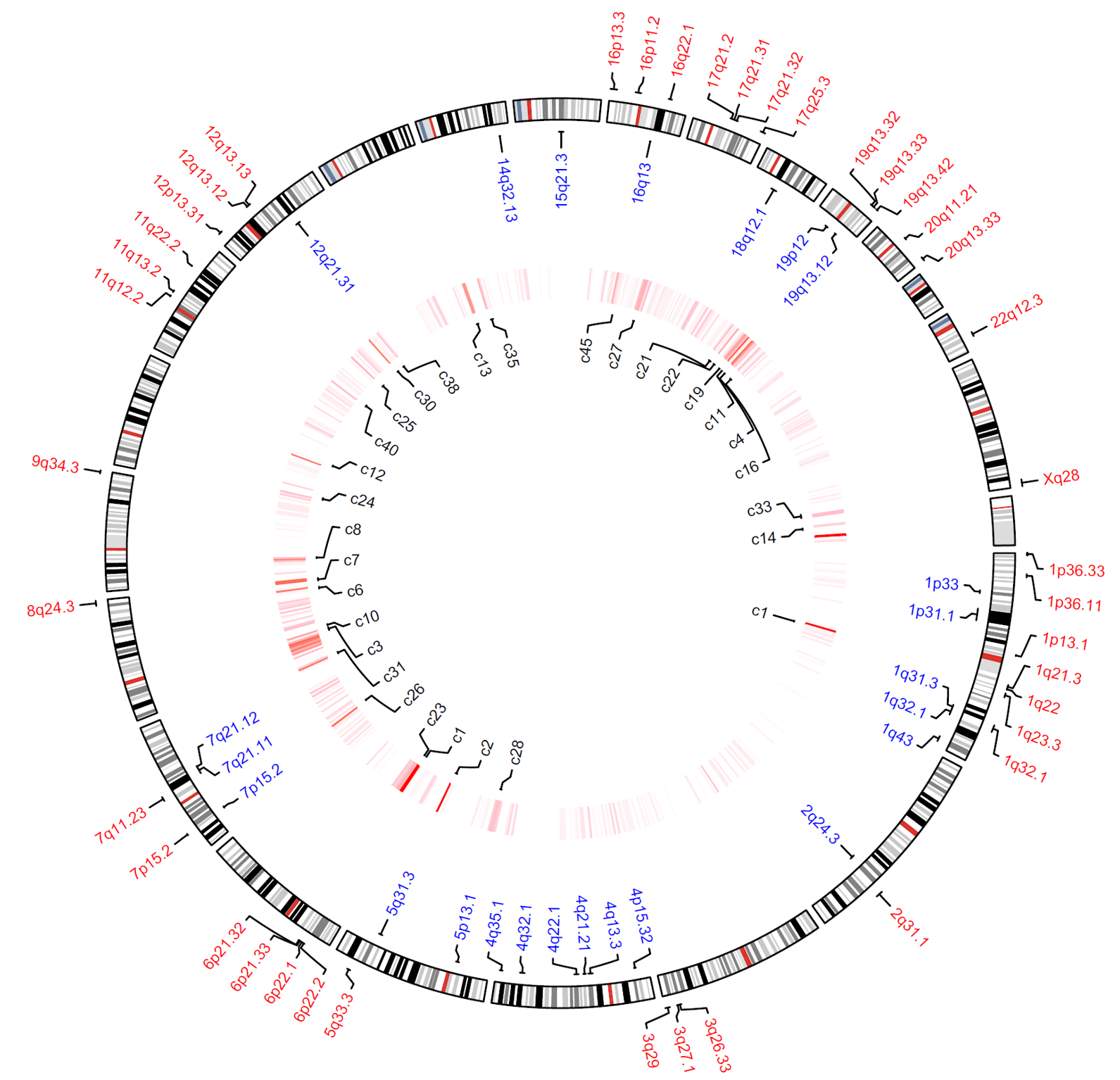


**Supplemental Fig. S5 The cytobands enriched for DEGs and conserved module clusters.** The Circos plot shows the locations of DEG-enriched cytobands and the corresponding module clusters enriched in these regions. The ideogram track displays the cytobands enriched for upregulated DEGs (red) and downregulated DEGs (blue). The inside heatmap track shows the cytoband enrichment of module clusters, with the red color intensity proportional to the number of cancer types where the module clusters are enriched in the cytobands. The top 30 module clusters with the most frequent cytoband enrichments are labeled.
